# Supplementary material for: Rehabilitation via HOMe-Based gaming exercise for the Upper limb post Stroke (RHOMBUS): a qualitative analysis of participants’ experience
Source: BMJ Open. 2024 Jan 20;14(1):e075821. doi: 10.1136/bmjopen-2023-075821 (PMC10806457; doi:10.1136/bmjopen-2023-075821)
Supplement: Supplementary data [file bmjopen-2023-075821supp001.pdf]

Supplementary Material 1: Interview Topic Guide

RHOMBUS Interview Topic Guide

Listed below are sample questions. The content is indicative. Questions will not necessarily be asked in this order nor phrased in the way presented here.

Consent

- Purpose of the interview:
  - to find out your views on the RHOMBUS study, how it went for you, whether it made any difference to you, and how we can improve it.
  - To learn what you thought about taking part in the research study.
- So it is important to be as honest as you can so we know what works and what needs to change. You don't have to answer any question, and can stop whenever you want.
- I'll record the interview so I can listen to you properly. What you say will remain confidential to the research team. Any quotes in reports of the study will be anonymised to protect your identity.
- Obtain written consent.

Preparing to take part in RHOMBUS

- Why did you volunteer to take part in this study?
  - What were you hoping for from taking part in the RHOMBUS study?
  - *Prompts*: goals/hopes
- Did you know enough about what was involved in the study before you took part?
- Did you have any concerns about taking part? If so, what were these?

Preparation for Intervention

- At the start of the study your received training on the NeuroBall. How did you find the training?
  Prompts:
    - What did you find helpful / unhelpful?
    - Did we take account of your own needs/ circumstances?
- You were also give an instruction handbook and a quick start guide, what did you think of these?

Impairments and limitations pre-intervention

- Going back to before you took part in the study and used the NeuroBall, can you reflect on the day to day use of your arm affected by your stroke?
  - *Prompts*: therapy at home, home exercise programme, upper limb activity levels historically and now

Using the NeuroBall

- How able were you to put on and use the device by yourself?
  - If help was needed what aspects did you need help with?
  - What would make it easier to put it on?
- In your own words can you tell me about your experience of using the NeuroBall as part of this study?

Prompt questions

- Was there anything in particular you liked / disliked about it?
- Which games did you like the most/least? Can you tell me why/what about it you liked/disliked?
- Did you like playing games on your own, or would you have liked to play against other players (stroke survivors or family or anyone)?
- Can you tell me about what you thought about the feedback it gave you?
- Do you think the NeuroBall helped with your arm and hand exercises?
- Did you track your progress on the device?
- Was it easy to pick which game you wanted to play and to use the menus?
- Did you suffer from any problems while using the NeuroBall (pain, motion sickness, headaches, falls, fear of falls, other concerns)?
- What did you think about the technical support offered?
- Do you think using the NeuroBall for 7 weeks had any effect on your arm? If so, can you describe the effect it had?

External Support

- During the study did you receive support from anyone/where else with using the NeuroBall?
- (If yes) what support did they give you with using the NeuroBall?

Acute use of the NeuroBall

- Thinking about someone who has recently had a stroke and is going through the same treatment pathway you did, do you think NeuroBall could be useful for them?
- Once trained, do you think you could have used the NeuroBall by yourself durng the time immediately after your Stroke?

Taking part in the research

- How did you find the assessment process?
- How did you find communication with the research team?
- When running the next phase of the NeuroBall studies there will be a chance of you receiving the NeuroBall but also a chance of not receiving a NeuroBall. Would this influence your decision to take part in the study?
- Would you be willing for us to contact you again in the future about this study or other studies related to stroke?
- Would you like to receive a report of this study?
- Is there anything else you would like to say about the NeuroBall or study?
