# Supplementary material for: Rehabilitation via HOMe-Based gaming exercise for the Upper limb post Stroke (RHOMBUS): a qualitative analysis of participants’ experience
Source: BMJ Open. 2024 Jan 20;14(1):e075821. doi: 10.1136/bmjopen-2023-075821 (PMC10806457; doi:10.1136/bmjopen-2023-075821)
Supplement: Supplementary data [file bmjopen-2023-075821supp002.pdf]

Supplementary Material 2: Participant Game Preferences

Game Preferences

NeuroBall Game/Activity Name	Brief description	Likes	Dislikes
**Scuba Diver Under the Sea**	This game is played by moving the NeuroBall up and down, therefore working on wrist flexion/ extension, elbow flexion/extension or shoulder flexion. The therapy game's protagonist is a diver who swims automatically forward and has to collect oxygen bubbles, so he does not run out of oxygen.<br>The patients move the diver vertically so that the diver collects these oxygen bubbles. Occasionally, the diver encounters treasure chests that can be opened by using the NeuroBall (by supination and pronation). The score increases the longer the diver swims without running out of oxygen and the more treasure chests he opens.	Interesting game. The different elements made it quite challenging and motivating. More than one type of hand movement was required. Motivating but not too difficult. Relaxing, rhythmical, low cognitive load. **P24 Linda:** ***Scuba Diving it was challenging as well as motivating for me (24, 1108). I felt that I could do more things with that, squeezing and moving my hand, er left and right as well, and forward and backward (25, 1113-1114). Scuba Diving was good as well because it was at the right speed [...] and I was able to grab the (Neuro) Ball and move it around (25, 1141-51). Scuba Diving, it was easier and nice and pleasant [...] (21, 932).***	Too easy and too slow. **P6 Mark:** ***[…] even the, the hard one like was much too easy for me (15, 647) […] Yeah, I used to get up to about 2 million or something, so... (15, 651) [...] Yeah, it was too slow (15, 656).*** **P18 William:** ***Er, I got a bit bored with playing that (18, 780)*** implying that the game was too repetitive or too long.
**Space Shooter**	To play this game the space ship is moved side to side on the screen, this can be done by supination and probation or flexion, extension at the wrist. A version of the popular	Offered a good range of hand movement. **P3 Ray:** ***I enjoyed that. That, that gives you plenty of hand movement. […] that was a nice game, good game, yeah. (18, 784-801).*** Similar to games played pre-stroke, **P18 William*****: [I***	Mixed findings, ***P5 Pam*** found it too easy, not enough levels of difficulty in the upper end, **P5 Pam: [Space shooter] that was quite... fairly easy. That got a bit annoying, I used to get annoyed with that (28, 1294-95). [...] you**

	Space Invaders game, adapted for neurological rehabilitation. The therapy game has 20 levels with enemy spaceships, asteroids and bosses, that come in waves and the patient has to destroy them all. The higher the level, the more difficult the therapy game becomes. The patients earn from one to three stars at the end of the game depending on their score.	***liked it] because when I was young, I used to play Space Invaders (15, 670).*** Offered the right amount of challenge, **P16 Ann:** ***I couldn't get the three stars on them so that was frustrating. Every time I tried it just wouldn't happen, so... But the other levels I sailed through. [...] So I loved that one (13, 561-73). I like the, the Space Invader one, I was like, "Oh I can do this, this is good, let's have another go. Oh let's have another go." (15, 656-57).***	**could go to the hardest one and that was just really as easy as the first few (30, 1365-66).** One participant found the level of difficulty too high, **P19 Sam: I tried but I just admitted defeat on that one (30, 1372). Like to actually progress. And it gets harder and harder, doesn't it? […] It's a little bit too hard. Well, not a little bit ... It's achievable (30-31, 1391-1395).**
**Holiday Dash**	This game is played by grasp and release of fingers to make the friends jump. This vacation or holiday themed game has the patient and four friends running along the beach. The runners will be running forward automatically.	Offered a physical challenge and an element of jeopardy which participants enjoyed. They were also able to improve their score over time if they persisted which they also liked. **P20 John:** ***Quite good, yeah, umm, because you have to jump the obstacles and catch the, umm, er, the rewards. And if you do it wrong you fell in the drink (6, 220-21). You had to squeeze […] to jump (29, 1321). I think that was good for you probably, the jumping (29, 1325).***	**P20 John** implied that the movements required to play were not complex enough: **the movements [for holiday dash] were, umm, forwards, backwards, sideways, squeeze and that was it (6, 231-32).** Two participants **Lina (P2)** and **Pam (P5)** found the squeeze action possibly too difficult, **P5 Pam: I didn't really like that one […]** ***'Cos the squeezing was... yeah, it was good for me but, you know, I didn't enjoy doing that as much (18, 784-93).*** **P5 Pam** also thought that the rewards and points were not clearly linked to game progression: ***And there wasn't enough, you know, rewards as you went through. You know, umm, the rewards were, umm, was it suntan cream, umm, all the different books to read and this sort of... [...] But, you know, they didn't have any effect on the game. [...] Winning the***

			rewards didn't have any effect on the game, or I didn't find out if it did [Laughing] (19, 822-33). I think I probably did earn more points but even that didn't seem to get you [any further] (19, 850).
**Pong**	In this game you act like a "goalie" by moving net up and down to stop the ball coming in. Wrist supination and pronation, flexion or extension can be used to do this. The patients control an in-game paddle by moving it vertically in a 2D environment. The patient selects a country and competes with other countries – each has a different level of difficulty. Patients are rewarded with bronze, silver, and gold medals based on their scores.	Participants enjoyed Pong because a) they liked football **(P2 Lina),** b) found the game challenging and they learned about arm control and accuracy **(P2 Lina, P19 Sam). P19 Sam:** ***if you moved fast you'd go shooting past the ball, so the ball, you know, goes in the goal […] But if you, if you control it slowly, which is good, it teaches you to do it slowly, you can, umm, bat the ball but if you do it too fast you go past the ball (6, 213-19).*** The sense of playing against an opponent was attractive to **P27 Elaine** as well as the sense of jeopardy (i.e. that there was something to lose), and the positive feedback of beating an opponent, **P27 Elaine:** ***Just you felt like you were playing against someone, I think that's what it was. And you're very…very smug if you beat 'em (10, 429-30). I found the football was a good challenge (10, 442). I think it motivated me. And that made it more enjoyable, you know, you was sort of…I was determined I wasn't going to lose (10, 454-55).***	Most of the participants who disliked Pong found it difficult to play **(P1 Iris, P16 Ann, P24 Linda, P28 Terry).** Also repeatedly losing i.e. failing was disappointing or unmotivating, **P1 Iris:** ***The Football one, that one was... I found it too difficult. Not too difficult, umm... Well I was losing all the time [laughs] and I thought, "Right, that's not for me." (17, 765-770).***
**NeuroMan**	An adaptation of the popular PacMan game for neurorehabilitation. The	Offered a lot of hand movement **(P6 Mark)** or an appropriate challenge i.e. quite a difficult	Most participants who didn't like playing PacMan found it too difficult **(P5 Pam, P16 Ann, P18**

	patients move the NeuroBall to control the NeuroMan, eating all the dots in the maze while avoiding the coloured ghosts. The have to use multiple movements of supination, pronation, wrist flexion and extension to move the NeuroMan.	game that participants found motivating **(P5 Pam, P19 Sam). P19 Sam: The one that I got better at was, umm, that Pac-Man one. I kept... Er, you know, they would get you, I was flummoxed and, er, let 'em catch me but after a while I knew how to outrun 'em. You know what I mean? (27, 1240-46).**	**William, P27 Elaine), P5 Pam:** ***You could have more levels […] where Pac Man […] is much slower and then the next level he's faster and then, you know, the third level is faster (24, 1084-93). I never got to more than one monster (24, 1105). The controls were difficult to go. You had to be perfectly in the middle to go left or right, yeah (25, 1114-15). [...] there didn't seem enough control on the Pac-Man (25, 1130). [...] it was difficult, yeah. You saw the arrow which... so, you know, told you which way you're going, but yeah [...] Again, you had to be perfect on the turning (25, 1140-45).***
**Solitaire**		**Only P20 John** spoke positively about Solitaire. His hand function may have made this game easier for him than for most of the other participants. **P20 John:** ***The Solitaire, it seemed more interesting but, umm, the movement required was quite difficult. You had to mark the card, pick it up (6, 232-33).***	Two thirds of participants did not play or reported that they did not enjoy playing solitaire. Six **(P2 Lina, P5 Pam, P18 William, P22 Bal, P23 Sue, P24 Linda)** found the game too difficult because it required quite precise control. **P5 Pam:** ***you had to have the ball perfect so you're going perfect, so you only had to be slightly off, and they would do the up thing all the time. [..] it was really good for control [laughing] [...] that was hard yeah, to start with (8, 314-328). I nearly finished it a few times actually, umm, but, yeah, I got fed up with it (laughs) before I did finish it (25, 1153-54). It was difficult to control, umm, and, umm, it took a bit long to get all the way, you know, from one place to another, umm, sometimes (26, 1159-60).***

			Participants **(P3 Ray, P5 Pam, P19 Sam, P22 Bal, P23 Sue, P24 Linda)** also reported difficulty understanding what it was they were supposed to do. They found the game uninteresting, due to a lack of momentum and the need to play for a long time before achieving something, so there were no easy wins, **P19 Sam:** ***I found it a bit tricky, that […] I just thought it was too slow and disjointed. I didn't keep the rhythm going, you know? […] It took too long (4-5, 137-54). You even showed me how to use that game 7 but I just couldn't, I couldn't get the gist of it at all. I think it, I think it's because the other games had momentum and, umm, something to achieve quickly, whereas on the last one, 7, the card game, you had to think about moving all the time, dragging that (12, 511-14).***
**Frenzy**	A octopus is crawling on the sandy floor, gobbling up a variety of shells and starfish. The octopus also needs to move quickly to avoid the bombs that are dropped and moving blobs, which result in its death. To control the octopus you have to move supination and pronation and wrist flexion and extension.	A challenging and interesting game that rewarded perseverance. **P2 Lina:** ***I like the Frenzy. […] It was hard though, but I persevered and I wanted to get my levels better, yeah (16, 622). […] because there's lots of things going on (15, 666).***	Most participants who didn't like playing Frenzy found it too difficult - too fast and difficult to control **(P5 Pam, P13 Steve, P25 Ed, P27 Elaine)**. P5 Pam and P27 Elaine thought more instructions were needed. **P27 Elaine:** ***I struggled with that game with the little octopus (6, 233). I really struggled with that, but I didn't…I couldn't find anything in the handbook that told me why I was struggling (6, 237-8). Yeah, it would go to the right … And then you couldn't get it back to the other side…or into the middle (6, 243-44). I didn't like, umm,***

			Frenzy, because I couldn't…couldn't do it. I suppose (18, 801-802).
